# Oral health providers’ views of oral health promotion in Jazan, Saudi Arabia: a qualitative study

**DOI:** 10.1186/s12913-023-09170-8

**Published:** 2023-03-06

**Authors:** Mosa Ali Shubayr, Estie Kruger, Marc Tennant

**Affiliations:** 1 International Research Collaborative, Oral Health and Equity, School of Human Sciences, The University of Western Australia, 5 Stirling Highway, Crawley, Nedlands, WA 6009 Australia; 2 Department of Preventive Dental Sciences, College of Dentistry, Jazan University, Jazan, Kingdom of Saudi Arabia

**Keywords:** Barriers, Jazan, Opportunities, Oral health, Promotion, Saudi Arabia

## Abstract

**Background:**

Oral health promotion (OHP) is a crucial aspect of dental care, as it aims to improve and protect oral health of individuals. This study aimed to qualitatively explore views of oral health providers in Jazan, Saudi Arabia, on their perceptions of their responsibilities for OHP, as well as the barriers and potential opportunities for implementing health promotion in dental practice.

**Methods:**

A convenience sample of 11 oral health providers from Ministry of Health (MOH) facilities were recruited and participated in virtual one-on-one semi-structured interviews, which were transcribed and analyzed using inductive thematic analysis with N-Vivo software.

**Results:**

The results showed that the providers recognized the significant role and responsibility of OHP in improving oral health. However, several barriers hindered their OHP efforts, including a lack of training, funding, time, and a lack of interest in OHP. Potential opportunities for improvement included increasing recruitment of new oral health providers and educators, developing more training programs for providers and the community, and expanding support in terms of finances and logistics.

**Conclusion:**

The findings of the study suggest that oral health providers are aware of OHP, but that both patients and organizations will need to shift their behaviours and perspectives for OHP to be successfully implemented. Further research on OHP in the Kingdom of Saudi Arabia (KSA) is needed to validate these findings.

## Introduction

Oral health is a critical aspect of overall health and well-being, as poor oral health can lead to a range of negative consequences, including pain, difficulty eating and speaking, and even systemic health issues [1]. Good oral health is important for individuals of all ages, with particular importance for children, as good oral health during childhood can have long-lasting benefits and prevent future oral health problems [2, 3]. Oral Health Promotion (OHP) is a public health effort to protect and improve oral health, including addressing issues such as periodontal disease, dental caries, and oral cancer [3]. OHP involves a range of activities, including education, prevention, and treatment, as well as development of policies and systems that support good oral health, such as water fluoridation, dental sealants, and availability of oral health care services [3]. Effective OHP requires the participation and support of various stakeholders, including governments, communities, and health care providers [3].

The importance of developing local dental opinion leaders who promote prevention-focused practices in clinics and help create peer networks, routines, and fee schedules [4]. However, there is a strong focus on downstream, curative treatments in dentistry, rather than preventive care dentistry [5–7]. A cross-sectional survey of 500 dentists in Saudi Arabia found that while there was adequate awareness of preventive care among dentists, more support from policy makers was needed [8]. Studies have highlighted the impact of oral health education programs sponsored by the King Salman Centre for Children’s Health in Riyadh, Saudi Arabia [9], which have shown positive results in students and suggest the importance of repeated and reinforced oral health education for lasting impact. Despite the availability of free dental healthcare in Saudi Arabia, a low percentage (12% of those over 15 years old) regularly visit a dentist [10], highlighting the need for increased oral hygiene education. The Dental Directorate of MOH Saudi Arabia has implemented several oral health awareness programs, including the National Oral Health Program for Primary School Children, Ante-natal Preventive Dentistry Program, Oral Health Program for Pre-school Children, Health Tents Campaign, Pit and Fissure Sealant Programs, Topical Fluoride Application Program, Oral Health Weeks, and Community Oral Health Education [11].

Despite the government’s efforts, the population’s awareness of preventive oral healthcare is limited for various reasons, including the absence of structured prevention and education programs, and a lack of awareness campaigns [12, 13]. Financial constraints can contribute to the low awareness levels among the population. Oral health providers have low awareness levels regarding preventive oral healthcare [14], which may be due to a lack of structured prevention and education programs and a lack of training in oral health [14, 15]. There is a disconnect between available knowledge on preventing oral diseases and information being provided in dental practices, dental schools, and community-based programs [16].

A Knowledge, Attitude, and Practices (KAP) survey is a research method that assesses the knowledge, attitudes, and practices of a group of people with respect to a specific topic [17]. It can be applied as both qualitative and quantitative research method. This type of survey is typically conducted using interviews or self-administered questionnaires and involves collecting and analysing non-numerical data, such as people’s opinions, beliefs, and experiences. KAP surveys are commonly used in the fields of public health and social science research to understand how a population comprehends and engages with a particular topic, and to identify any gaps in knowledge or misunderstandings.

Oral health care providers can play a role in promoting positive oral health attitudes and behaviours in society by acquiring necessary skills and knowledge for practicing OHP. These skills and knowledge can be obtained through training and experience in OHP and prevention. Oral health care providers can contribute to positive changes in their own behaviour and approach towards promoting oral health [14, 18].

This qualitative study aimed to explore views of oral health providers in Jazan on oral health promotion, including their perceived challenges and successes in this area. By understanding the perspectives and experiences of these providers, it may be possible to identify ways to improve oral health promotion efforts in Jazan and other similar communities. Results of this study can be used as a starting point for future research on how Saudi Arabian oral health providers can promote oral health. Understanding the views of oral health providers on oral health promotion is important for developing effective and sustainable OHP efforts [3].

## Materials and methods

This study used a qualitative research method to examine oral health promotion among oral healthcare providers in the Jazan region. It included semi-structured, online in-depth interviews with a convenience sample of dentists working in MOH primary healthcare centres and hospitals with a dental component. Ethical approval was obtained from the Institutional Review Board at Jazan University’s College of Dentistry (REC41/5/133) and the Human Ethics Board at the University of Western Australia (RA/4/20/6235). The study did not include oral healthcare providers who worked in private clinics.

It is difficult to estimate the number of oral healthcare providers operating in the Jazan region, as the MOH’s most recent statistics from 2020 do not separate oral health hygienists and dental assistants as separate professional groups [19]. However, in 2020, there were 264 dentists in Jazan, spread across 168 primary healthcare centres and 19 hospitals in 17 governorates [19].

This study utilized the qualitative method of KAP survey as the theoretical framework to explore oral health promotion among oral healthcare providers in the Jazan region [17]. The thematic analysis approach has been widely used in medical literature to identify misconceptions, knowledge gaps, and barriers to behavioural change in KAP surveys [20].

### Recruitment of participants

Potential participants for this study were identified using the Internet, with “dentists,“ “dental assistants,“ “dental hygienists,“ “Jazan,“ and “Jizan” as keywords in the search. Initial invitation and information sheets were sent to selected potential participants through their personal accounts on Facebook, Twitter, WhatsApp, LinkedIn, ResearchGate, and Google Scholar. A reminder email was sent to those who did not respond after a week, but no third reminder was sent to avoid overburdening recipients with emails. Those who responded and agreed to participate in the study submitted an information sheet with a consent statement, including their email address and Zoom account number (if available). The best time for interview was selected to suit the participant, and a Zoom link provided via email. Permission was requested to record the virtual interview as audio or video (at the participant’s request), and those reluctant to be audio or video recorded were excluded from the study.

### Study instrument

The questions for the study were developed in English and based on previously published research [14, 21] and a comprehensive literature review. They focused on knowledge, attitudes, and practices of the participants with regards to oral health promotion, including their participation in oral health activities and their views on responsibilities and skills needed to promote better oral health in the general population. Questions addressed communication and collaboration between dental healthcare professionals and organizations regarding oral health promotion.

To ensure trustworthiness of the study, the criteria established by Lincoln and Guba [22] were followed, which included credibility, dependability, confirmability, and transferability. To improve credibility, clarity, and accuracy of the interview questions, the questions were reviewed by two dental public health faculty members at Jazan University and their recommendations were applied. To ensure dependability and confirmability, three pilot interviews were conducted with oral healthcare providers in Jazan. The initial themes were determined from the pilot interviews and use of the KAP framework ensured transferability of the study results.

### Data collection

Data collection for the study involved conducting online, semi-structured in-depth interviews with 11 oral healthcare providers working in MOH facilities. The interviews were conducted in English between July and December 2021.

The interviews were conducted at a suitable location where the participants were not at risk and there was no intimidation or coercion. Participants provided informed consent before the study began by clicking on a button after reading a consent paragraph that explained the voluntary nature of the study, nature of their participation, and methods for maintaining anonymity. The interviews began with an introduction that briefly described history of the research project, objectives of the interview, topics to be discussed, and estimated length of the interview. Permission was sought from each participant to record the interview, including questions on their knowledge, attitude, and practice of oral health promotion.

At the end of the interview, main points discussed were summarized, and participants were asked if they had any additional comments. The concept of data saturation was implemented during the interviews, which has been widely used in qualitative research related to oral health [23, 24] and has been extensively reviewed in the research community [25]. Participants were asked to reflect on their responses during the interviews until data saturation was reached or no new information was obtained. Each interview session lasted an average of 60 min.

### Data analysis

The data collected for this study were analyzed individually and then compared to evaluate the participants’ perspectives and the researcher’s observations. To ensure participants’ privacy, all personal details were removed before entering the data for analysis. The interview data were transcribed and compared to the original audio recordings to ensure accuracy. Two researchers, both dental public health professionals, were reviewed the content and initial coding based on the interview objectives. The NVivo 20 software package [26] was used to manage the data. The data collected from participants and the theoretical framework were used to create codes and categories. However, during the analysis process, a thematic analysis approach [27] was employed to explore connections between the collected data and the specified research framework of this study. The KAP framework was used to generate the initial codes, which were then used to identify and refine themes by reviewing all compiled extracts for each topic to ensure that the data within them followed a consistent pattern.

## Results

A thematic analysis of data on OHP identified three main themes. The first theme focused on the responsibility of healthcare providers to promote oral health among their patients and in the community. The second theme addressed the barriers and challenges that providers faced in their OHP practice and within the community they serve. The third theme consisted of providers’ recommendations for improving OHP in their clinics, workplaces, and community. These themes were captured in Table 1.

**Table 1:** Description of themes identifies in the thematic analysis of participants’ responses

Themes	Sub-themes	Theme Summary
Responsibility	1. Knowledge 2. Skills	Health education is regarded as the most important aspect of oral health promotion in dentistry. This focus on behavioural techniques and information dissemination could be linked to dental professionals’ believing they are more skilled in health education than in other health promotion strategies.
Barriers and challenges	1. Courses 2. Funding 3. Time 4. Health facility type 5. Personal	Due to the dental care funding scheme, clinical work takes precedence. Governments do not provide enough resources to promote oral health. Because they do not have enough money, dentists do not have much time to spend on health promotion. In the regions, there is variability in OHP practice across hospitals and Primary Healthcare Center (PHC). Patients and dental office managers place a low value on the necessity of health promotion.
Recommendations	1. Evidence-based training 2. Increase the number of providers 3. Tele dentistry	Awareness activities for dental professionals are needed to let them know how important it is for them to offer these services in their health settings. Overcome the paucity of time and lack of awareness about O among practitioners by offering health educators or newly graduated dentistry students. Tele-dentistry has been identified as essential in improving OHP among the region’s people.

### Responsibility toward OHP

The results of the study indicated that participants believe that OHP is the responsibility of oral health providers. This is reflected in their emphasis on concepts such as “prevention is better than cure,“ regular check-ups, and the importance of training dentists in OHP. Participants also emphasized the benefits of OHP, including reduced treatment costs, improved success rates of treatment, and the communal responsibility of dentists to promote good oral health in their communities. Overall, the results of the study highlight the importance of oral health providers taking a proactive approach to promoting good oral hygiene and educating their patients on the importance of maintaining good oral health.

Participant 4: *‘I believe that it’s one of any dentist’s responsibilities. Today, we do not only have to provide treatment services; if we can provide educational or awareness services, we will decrease the treatment services we provide. These treatment services get us tired as dentists, clinics, costs, supplies… We need to apply the quote “prevention is better than cure”. If there is actual prevention, we will decrease treatment costs, dental problems, ongoing patient visits, sessions that take so much time, and problems with these services. All of these will be decreased to prevent every certain period or check-up every 3–6 months.’*

Participant 5: *‘As I said. We are responsible and have to play a role in our community to benefit others. I tell the patients about oral health promotion for two reasons; first, it is good for them, and second, it’s my responsibility. I do my best to raise their awareness. So, I tell them everything; how to brush their teeth, when they should follow up, ask them whether they use dental floss, and other things like preventing them from using toothpicks, etc.’*

Participant 11: *‘Yes, it’s very important for me. I have videos uploaded on the clinic’s laptop. I open these videos to each patient to show them the correct way to brush their teeth. I have a model too because it has a good result with the health promotion for the children, and it differs greatly in their dental treatment. Even during restoration, if the patient has bad oral hygiene, the successful rate will not be high, so I have to explain to the patient to improve the oral hygiene to increase the rate of successful treatment.’*

Participant 3: *‘Currently, I don’t say that I have the required skills, but I have the skills that get the job done. I can attract the patient’s interest to listen carefully to what I say.’*

Participant 10: *‘To give lectures and so, no. I don’t have such skills. But, to help raise my patients’ awareness in the clinic, I do it usually.’*

### Barriers and challenges related to OHP

It is clear from these participants’ descriptions that there are several barriers to providing OHP to patients. These barriers include a lack of training in OHP, a need for regulatory authorities to develop materials and guidelines for patient education, and a lack of time and interest in regular check-ups and seminars among the community. These barriers can make it difficult for dentists to effectively promote good oral hygiene and educate their patients on the importance of maintaining good oral health. It is important for dental professionals and regulatory authorities to address these barriers in order to improve the effectiveness of OHP efforts.

The opinion related to the training received by the dentists regarding OHP are quoted below:

Participant 5: *‘From our studying in the college and through researching. There are some cases we search to know more about over the years through Google search, specialists, research, studies, etc. But not so much, to be honest.’*

Participant 10: *‘From university studies. Also, from campaigns we were doing in the university and then the campaigns we do with the centre for diabetes and so. All of our campaigns are to educate people on how to care for their teeth and their oral health. We don’t speak about curing or any deep information. So, I gain my knowledge from the university and the campaigns we do in the centres.’*

The opinion related to hospitals not providing adequate facilities and materials, along with the lack of support are quoted below:

Participant 3: *‘Sure, the hospital will provide a place for such activities and will provide whatever they can. But to be honest, the hospital won’t be able to provide most of the tools. Sometimes we contact the centre to send us toothpaste packages, which is like struggling as they sometimes send us what we need and sometimes delay on this. The same is for the leaflets and this is because there is no competent authority for such awareness. We even have to use old tools sometimes.’*

Participant 8: *‘Yes. To be honest, there is no support for these programmes at the level of the region.’*

Participant 1: *‘For me, I need support to start. Whenever I try to participate in Gulf Cooperation Council (GCC) Oral Health Unified week, I struggle to have the materials I need to educate the patients. So, there should be a competent authority.’*

The opinion regarding the lack of time in hospitals and no dedicated efforts are highlighted below:

Participant10: *‘It depends. If my shift is in the morning, I have the opportunity to participate in the evening. But if my shift is in the evening, I can’t participate except in-the-centre activities like making a stand or a table and receiving the kids to check up for free. I prefer the in-the-centre activities more than the outside activities.’*

Participant 3: *‘While working, no. I don’t have any time to educate the patient.’‘I don’t think so, as I am full-time in the hospital’*.

The opinion regarding the lack of interest amongst people to attend seminars or have discussions with the dentists are quoted below:

Participant 3: *‘The whole community. There is no interest in seminars and lectures by the community. I think we need to determine the best way of awareness for the community. They don’t care for seminars and lectures.’*

Participant 8: *‘People get bored of the theoretical information and lectures. A few may attend these lectures if we announce that we’re going to give a lecture. Sometimes, we do an activity which is a lecture, and we announce it a day or two days before it’s time. To have many attendees, we have to obligate them. If attending is optional, no one will attend. However, if we prepare brochures, models, or gifts during GCC Oral Health Unified Week, people will attend it. So, people aren’t interested in attending lectures.’*

Participant 7: *‘The patient doesn’t have the education or the awareness that could encourage them to visit the dentist every 6 months, to scale his teeth, or to check whether they have caries or anything. He just goes to the dentist because of the pain. There is neither awareness nor education that could let him visit the clinic just to check up. So, I think that the level is not good.’*

### Recommendation and Opportunities for oral health promotion

Oral health professionals who work directly with patients have a unique opportunity to promote good oral hygiene in society. Providing evidence-based knowledge and education to patients, as well as encouraging regular check-ups, are important strategies for promoting oral health. During the interview, the hiring of health educators and the setting up of oral health promotion clinics were mentioned as effective approaches. The use of virtual clinics was also highlighted as a way to make it more convenient for patients to access oral health education and services.

Participant 11: *‘To be evidence-based. Okay? And to be scheduling. Each period, there should be frequent programmes, not only once. Also, following up of the evidence is required. For example, I did a dental diagnosis for a group of people, so I should follow up with them after time. Of course, they should be evidence-based to be effective, in my opinion. If I exposed some people to diagnosis, I should follow them up, especially Pedodontics or children. I should follow them up to measure the effectiveness, before and after measurement or feedback, organize programmes, including evidence-based programmes discussing multiple effective ways.’*

Participant 6: *‘we need to raise dentists’ awareness. Then we can spread it in the community. It’s so important to start performing programmes. There are dentists, and I’m one of them, who see that it’s not important to that extend or that there are a lot of programmes that we don’t know anything about. This is the first point. If we have enough dentists who understand the situation well, we can solve the other problems.’*

Participant 11: *‘At the level of my workplace, it’s so necessary to hire health educators. It’s necessary. Sometimes because the dentist has a lot of patients, he can’t teach all patients, or he can’t sit and explain to all patients. Sometimes in some emergency cases, patients’ numbers increase, which can further limit the time available for providing education. This is the first point at the level of the workplace. It’s supposed to have a health educator when the patient gets in the doctor’s clinic or before it, he’s referred to the health educator.’*

The need for support from health organizations to increase the availability of OHP related materials and OHP related activities in highlighted in the quotes below:

Participant 3: *‘Yes, to have specific clinics for enhancing oral health promotion everywhere. Also, these clinics should be the main resources for fluoride and materials we need and to launch several initiatives for schools once or twice a month.’*

Participant 1: *‘We need to form a competent authority to organise, coordinate and facilitate the process for us. To find specific places for such activities. all of these are necessary and make it easy for us to perform good things. Also, to do these activities more than once a year, not just one time. To do them from time to time, on an ongoing basis.’*

Participant 2: *‘They are the solution of the obstacles we mentioned; like having good materials, working devices, support from the health organization of the region or district, nursing, and reducing the number of patients to be able to educate each patient without being pressured.’*

These opinions strongly suggest the importance of tele-dentistry and virtual clinics in providing dentists with the time to promote OHP.

Participant 11: *‘to have support for the dentist to educate the patients or to use the virtual clinic. This is at the level of the workplace.’*

Participant 9: *‘Virtual communication. Dental services are provided by the Ministry of Health. We can use these virtual clinics for oral health promotion enhancement. Currently, If I have a patient and I don’t have time to teach him, I refer this patient, or I give him an appointment in a virtual clinic. Virtual clinics are communicative clinics in the same centre. For any patient whom I couldn’t advise after the delivery of his crown or bridge, and I want to improve his oral hygiene, I give him an appointment and write in his file all the weakness points he needs to improve and I refer him or give him an appointment in the virtual clinic after getting acceptance from the patient.’*

## Discussion

Despite the importance of oral health providers in promoting oral health, this study is the first in Jazan, Saudi Arabia, that investigated OHP among oral health providers. In addition, the study examined the barriers and challenges faced, recommendations for improvement, and opportunities for enhancing OHP in the region. Oral health care providers have the important task of promoting oral health and disseminating preventive information to the numerous patients they see in their clinics on a daily basis [28]. The participants in this study had a positive attitude towards improving OHP in Jazan and recognized their responsibility for educating and improving knowledge about oral health among their patients and the surrounding community [14, 29]. This is an improvement from previous studies in Saudi Arabia that found negative attitudes among participants [30] or only fair attitudes towards OHP [14]. However, the results of this study contradict a previous study where dentists did not see prevention as their role because their efforts did not impact oral health or patient behaviour [31].

The study also found that oral health providers need more training and education to improve their knowledge and skills in OHP. Limited time for health promotion and a lack of funding and support for OHP activities [32, 33] were identified as barriers to OHP. Health promotion activities should be supported financially and logistically [34] in order to increase awareness about good oral hygiene in the community. Organizations often prioritize treating diseases over preventing them, potentially because oral health promotion does not produce immediately measurable results. The government and policymakers should focus on implementing policies that ensure health facilities consistently engage in activities aimed at promoting oral health in all regions of Saudi Arabia.

Factors that influence OHP among providers in the region were also highlighted in this study. A lack of understanding and knowledge about OHP can affect the message that oral health providers convey to their patients [14]. Gaining knowledge and skills in OHP can lead to positive changes in the behaviour of health care providers towards OHP [20]. Studies among dental workers show that providers who receive more continuing education in OHP are more engaged in OHP [14, 35]. In addition, since some of the study participants received their knowledge about OHP from their undergraduate degree, it should be emphasized in dental school curricula, particularly in dental community courses that effectively address OHP and disease prevention for the entire population.

Collaboration between public and private organizations should be established to improve OHP and increase awareness of good oral hygiene among the population [36]. Public-private partnerships can enhance the impact of outreach programs by addressing social, educational, and clinical aspects, and can also reduce the burden on public organizations, facilitate the adoption of new technologies, improve public perceptions of OHP, and provide access to better training programs [37].

According to the study participants, a lack of time can prevent dental providers from participating in OHP programs and activities. Tele dentistry was identified as a potential solution to improve OHP in the region. Participants also emphasized the importance of health educators, who can help patients understand the importance of oral hygiene alongside dentists. Mobile apps could also be effective in promoting oral health [38]. To address the issue of limited time, existing dental clinicians may be compensated for participating in OHP programs outside of their regular work hours, or new dental health educators or hygienists can be recruited. There is also a need for the government to create realistic employment opportunities for Saudi dentists, as there are currently 900 unemployed Saudi dentists out of a total of 5287 licensed general dentists [39]. The MOH could establish a temporary paid program for recent graduates (lasting 6–12 months) to provide preventive and treatment services in urban and rural areas.

There were reported differences in OHP practices between hospitals and primary health care centres (PHCs), with PHCs tending to focus more on OHP than hospitals [14]. However, this may not always be the case, and the inconsistency could be due to various factors such as the management and understanding of the importance of OHP within the hospital. The KSA government through the MOH has also implemented public health promotion programs in recent years in an effort to control and prevent oral diseases [11], which may have contributed to the difference in OHP practices between hospitals and PHCs.

The study participants also noted a lack of community engagement in OHP, potentially due to a lack of awareness, financial barriers, or irregular access [31]. This is consistent with the findings of a study conducted in Jazan, where a third of participants believed that a dentist should be visited only when experiencing pain [40]. This lack of engagement may contribute to the high rate of dental cavities in the region, which has increased from 68 to 96% in recent years [41, 42]. Children, adults, and elderly all have a high prevalence of dental caries [41, 42]. Participants indicated that the public and patients are not interested in OHP because they do not see immediate results or benefits compared to treatment.

Dental providers must also have the necessary skills and model good oral health behaviours for the communities they serve [43, 44]. Dental health professionals can significantly impact communities by educating them about oral health and encouraging the adoption of healthy behaviours and the reduction of unhealthy ones [45]. However, participants reported a lack of skills in educating the public about oral health, and some reported using tools to promote oral health among their patients.

### Strengths and Limitations

This study has several strengths that should be considered when interpreting its results. The use of a semi-structured interview method allowed oral health professionals to share their thoughts and experiences related to oral health promotion in dental clinics and the community in a free-flowing manner, which provided a wide range of opinions. The study also focused on perspectives of oral health providers (dentists), who play a crucial role in OHP but have not been extensively studied in the past. This is particularly relevant for informing provider planning and resource allocation for OHP needs.

However, the study has some limitations that should also be explored. One limitation is that it did not include other types of oral health providers, such as hygienists and assistants, who may have different perspectives on OHP. This may be because there are a few number of these providers in Jazan [46] and it was difficult to find them online. Another limitation is that the study did not collect demographic information about the participants, so it is not possible to determine if the characteristics of the participants influenced the data [32]. The decision to not collect this information was made for several reasons. Firstly, to ensure that the participants felt comfortable and anonymous during the study, as collecting demographic data may have compromised this. Additionally, the authors were concerned that including demographic data could introduce bias or stereotyping into the analysis of the results. Despite this limitation, the authors believe that the study still makes a valuable contribution to the field. The low response rate (45 oral health providers were initially contacted) may also be a limitation, as it represent a sample that is not representative of all oral health professionals. This low response rate may have been due to time constraints, lack of incentives for participation, and difficulty in reaching respondents through the internet. It is also possible that the results of the study are biased because the respondents may have particularly strong opinions or enthusiasm for health promotion. A larger sample size would have likely increased the validity of the study’s results, as it would have allowed for a more diverse and representative sample of oral health professionals. However, it is worth noting that similar study with small sample sizes of around 25, are published and have contributed to important scientific discourse [33].

It is important to note that these findings of this study may not be generalizable to other populations or settings, as the study was conducted in a specific geographic location with its own unique healthcare system and cultural context. More research is needed to understand how these findings may apply to other contexts and to explore the perspectives of other oral health professionals. Despite these limitations, the results of this study provide valuable insights into the barriers and facilitators of OHP from the perspective of oral health professionals and can inform development of interventions to improve the effectiveness of OHP efforts.

## Conclusion

The results of this study provide valuable insights into the views of oral health providers on OHP in Jazan, as well as the barriers and challenges they face. The providers in this study recognized the significant role and responsibility of OHP in improving oral health status through education and knowledge dissemination to their patients and the community. However, they identified several barriers to their OHP activities, including lack of training, funding, time constraints, and lack of interest in OHP. Potential opportunities for improvement could include increasing the recruitment of new oral health providers and educators, developing more training programs for providers and the community, and providing greater support, whether financial or logistical. Public and private partnerships may also be considered to improve and facilitate OHP and prevention activities. Further research on OHP in the KSA is needed to confirm these findings.

## Data Availability

All data that support the results of this study are with the corresponding author [M.S.] and can be made available upon reasonable request.

## Abbreviations

KSA: Kingdom of Saudi Arabia
MOH: Ministry of Health
PHCs: Primary Healthcare Centres
WHO: World Health Organization
OHP: Oral Health Promotion
